# Single‐cell RNA sequencing technologies and applications: A brief overview

**DOI:** 10.1002/ctm2.694

**Published:** 2022-03-29

**Authors:** Dragomirka Jovic, Xue Liang, Hua Zeng, Lin Lin, Fengping Xu, Yonglun Luo

**Affiliations:** ^1^ Lars Bolund Institute of Regenerative Medicine Qingdao‐Europe Advanced Institute for Life Sciences Qingdao China; ^2^ BGI‐Shenzhen Shenzhen China; ^3^ Department of Biology University of Copenhagen Copenhagen Denmark; ^4^ Nanjing University of Chinese Medicine Nanjing China; ^5^ Department of Biomedicine Aarhus University Aarhus Denmark; ^6^ Steno Diabetes Center Aarhus Aarhus University Hospital Aarhus Denmark

**Keywords:** atlas, big data, precision medicine, regenerative medicine, RNA sequencing, single cell

## Abstract

Single‐cell RNA sequencing (scRNA‐seq) technology has become the state‐of‐the‐art approach for unravelling the heterogeneity and complexity of RNA transcripts within individual cells, as well as revealing the composition of different cell types and functions within highly organized tissues/organs/organisms. Since its first discovery in 2009, studies based on scRNA‐seq provide massive information across different fields making exciting new discoveries in better understanding the composition and interaction of cells within humans, model animals and plants. In this review, we provide a concise overview about the scRNA‐seq technology, experimental and computational procedures for transforming the biological and molecular processes into computational and statistical data. We also provide an explanation of the key technological steps in implementing the technology. We highlight a few examples on how scRNA‐seq can provide unique information for better understanding health and diseases. One important application of the scRNA‐seq technology is to build a better and high‐resolution catalogue of cells in all living organism, commonly known as atlas, which is key resource to better understand and provide a solution in treating diseases. While great promises have been demonstrated with the technology in all areas, we further highlight a few remaining challenges to be overcome and its great potentials in transforming current protocols in disease diagnosis and treatment.

## Graphical abstract

1. This review provides a concise summary of the single‐cell RNA sequencing technologies.
2. Overview and guidelines for planning experimental procedures are presented.
3. Bioinformatics tools for scRNA‐seq data analysis are thoroughly discussed.
4. Applications and further development of scRNA‐seq technology are highlighted.

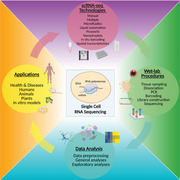

## 1 THE RISE OF SINGLE‐CELL RNA SEQUENCING TECHNOLOGY

Humans are highly organized systems composed of approximately 3.72 × 10^13^ cells of various types forming harmonious microenvironments to keep proper organ functions and normal cellular homeostasis.^1^ Living cells were observed for the very first time in the 16th century, and since then many signs of progress and advancement of new technologies and methods evolved from elementary to profound. Although the first microscope invented by Zacharias Janssen and Hans Lippershey in the late 16th century enabled Robert Hooke and Anton van Leeuwenhoek to spot the first living cell in the 17th century, it took almost two centuries to re‐define cells not only as of the structural but also functional unit of life.^2^ Since then, various experiments and methods were conducted for the purpose of better understanding and investigating cells in heterogeneous multicellular systems.^3^, ^4^ Despite that tremendous and revolutionary discoveries in cell biology field have been made, the heterogeneity of cells remains to be further revisited. Almost all cells in the human body have the same set of genetic materials, but their transcriptome information in each cell reflects the unique activity of only a subset of genes. Profiling the gene expression activity in cells is considered as one of the most authentic approaches to probe cell identity, state, function and response. Huge technological breakthroughs have been made in the single‐cell transcriptomics during the last decade. With single‐cell RNA sequencing, it is now possible to analyse the transcriptome at single‐cell level for over millions of cells in a single study. This allows us to classify, characterize and distinguish each cell at the transcriptome level, which leads to identify rare cell population but functionally important.

The first conceptional and technical breakthrough of the single cell RNA sequencing method was made by Tang et al. in 2009, which sequenced the transcriptome of single blastomere and oocytes.^5^ The concept and technology brought by this study open a new avenue to scale up the number of the cells and make compatible high‐throughput RNA sequencing possible for the first time. Since then, an increasing number of modified and improved single‐cell RNA sequencing technologies were developed to introduce essential modifications and improvements in sample collection, single‐cell capture, barcoded reverse transcription, cDNA amplification, library preparation, sequencing and streamlined bioinformatics analysis. Most importantly, cost has been dramatically reduced, while automation and throughput have been significantly increased. All these steps branch into more matured scRNA‐seq methods, but the concept of the scRNA‐seq remains the same. This review provides a comprehensive and concise overview of the single cell technology development from its early stage and library constructions and its challenges and data acquisition that transform our understandings of RNA transcriptions into data output. We also discuss applications of scRNA‐seq, the potential of the scRNA‐seq in spatial transcriptomics, cell atlases and future perspectives.

## 2 HIGH THROUGHPUT SINGLE‐CELL RNA SEQUENCING TECHNOLOGY: EXPERIMENTAL PROCEDURES

**FIGURE 1:**
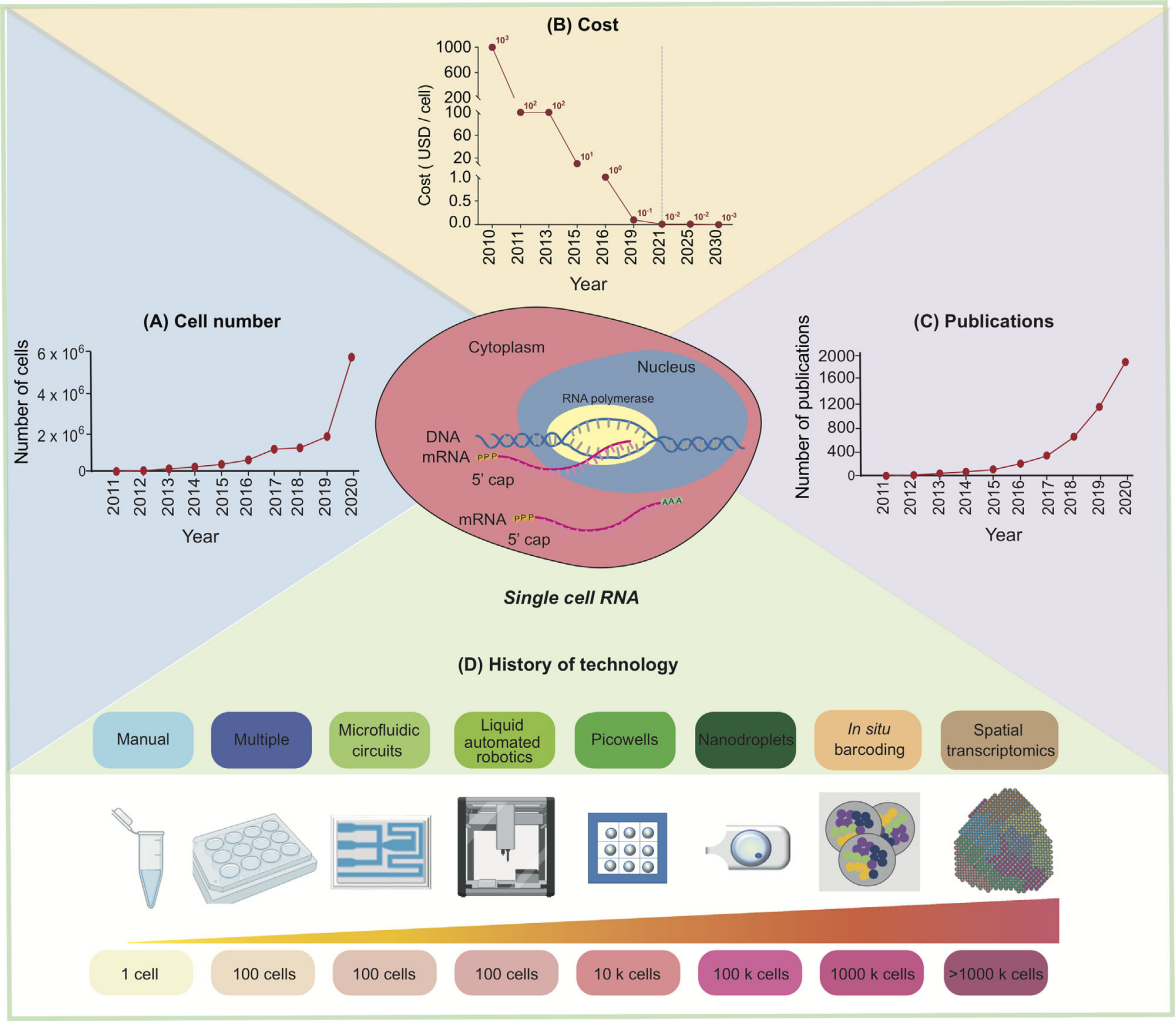
Development of single‐cell RNA sequencing technology. With the technological advances in single‐cell RNA sequencing (scRNA)‐seq, (A) the number of analyzed cells increased, (B) the cost (in US dollar) was exponentially reduced, (C) the number of published papers increased and (D) the history of technology evolution in the last decade using more sophisticated, accurate, high throughput analysis was achieved. Part (D) is created with icons from BioRender with license for publication

The throughput of scRNA‐seq increases from a few cells per experiment to hundreds of thousands of cells, where the cost has been tremendously reduced. As such, scientific publications using the scRNA‐seq technology method each increases yearly due to fast and accurate scRNA‐seq technologies such as microfluidic‐ microwell‐, droplet‐based, in situ barcoding and spatial transcriptome analysis as summarized and illustrated in Figure 1.^6^, ^7^, ^8^, ^9^, ^10^ In this section, we focus on highlighting a few key technological and experimental steps in high throughput single‐cell RNA sequencing technologies.

**FIGURE 2:**
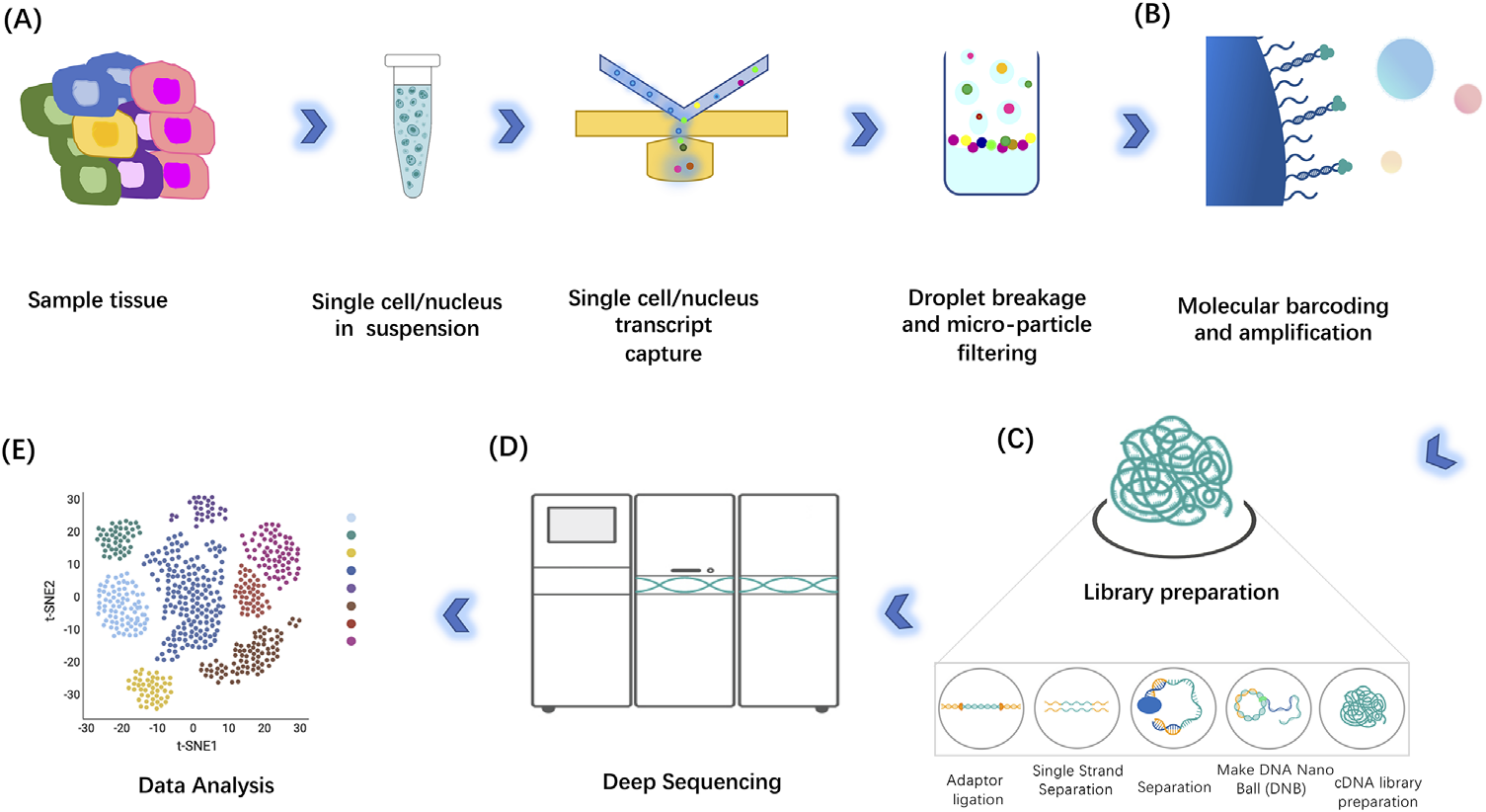
An overview of the single‐cell RNA‐sequencing procedures. (A) Isolation of the cells from tissue samples and capturing of the single cells, wrapping of each individual cell with a bead inside a nanoscale droplet (each bead contains unique molecular identifiers), (B) barcoding and amplification of complementary DNA (cDNA) and (C) library preparation procedure. After single‐cell RNA sequencing (D), the snapshot data would be analyzed to present and classify the landscape of gene expression in cells of a heterogeneous population (E). Illustrative figure in (E) is generated with BioRender with license for publication

The procedures of scRNA‐seq mainly include single‐cell isolation and capture, cell lysis, reverse transcription (conversion of their RNA into cDNA), cDNA amplification and library preparation (Figure 2).^11^, ^12^ Single‐cell capture, reverse transcription and cDNA amplification are the most challenging parts among the library preparation steps. With the development of many sequencing platforms, RNA‐seq library preparation technologies have also presented a rapid and diversified development. Thus, it is important to know the features and applications of different single‐cell RNA sequencing library preparation methods, in order to make appropriate choices in scientific research and better apply these techniques to clinical applications.

Single‐cell isolation and capture is the process of capturing high‐quality individual cells from a tissue, thereby extracting precise genetic and biochemical information and facilitating the study of unique genetic and molecular mechanisms.^13^ Traditional transcriptome, epigenome or proteome from bulk RNA/DNA samples can only capture the total level of signals from tissues/organs, which fail to distinguish individual cell variations. The single‐cell isolation and capture methods are largely different depending on the organisms, tissues or cell properties.^14^ Cell isolation can be accomplished by isolating whole cells, cell‐specific nuclei or cell‐specific organelles, and even by separating the desired cells expressing specific marker proteins.^15^ The most common techniques of single‐cell isolation and capture include limiting dilution, fluorescence‐activated cell sorting (FACS), magnetic‐activated cell sorting, microfluidic system and laser microdissection. The key outcome of single capture, and particularly in high throughput, is that each single cell is captured in an isolated reaction mixture, of which all transcripts from one single cell will be uniquely barcoded after converted into complementary DNAs (cDNA).

However, the scRNA‐seq has gradually revealed some inherent methodological issues, such as ‘artificial transcriptional stress responses’. It means that the dissociation process could induce the expression of stress genes, which lead to artificially changes in cell transcription patterns. This has been confirmed by a number of experiments. Brink et al. found that the process of protease dissociation at 37℃ could induce the expression of stress genes, introduce technical error and cause inaccurate cell type identification.^16^ Adam et al. also found that dissociation at 37℃ can cause ‘artificial changes’ of cell transcriptome, resulting in inaccurate results.^17^ Dissociation of tissues into single‐cell suspension at 4℃ has thus been suggested to minimize the isolation procedure‐induced gene expression changes. Single‐nucleus RNA sequencing (snRNA‐seq) is an alternative single‐cell sequencing method. Instead of sequencing all the mRNA in the cytoplasm of cells, scRNA‐seq only captures the mRNAs in the nucleus of cells. The snRNA‐seq solves the problems related to tissue preservation and cell isolation that are not easily separated into single‐cell suspensions, applicable for frozen samples, and minimizes artificial transcriptional stress responses as compared to scRNA‐seq.^18^ SnRNA‐seq becomes very useful in many tissue types, such as muscle tissue,^19^ heart,^20^ kidney,^20^ lung,^21^ pancreas^22^ and various tumour tissues.^23^ It is particularly applicable in brain tissues, which are difficult to be dissociated to obtain intact cells. Grindberg et al. demonstrated that single‐cell transcriptomic analysis can be done using the extremely low levels of mRNA in a single nucleus of brain tissue.^24^ Therefore, several compelling potential benefits of the method emerge: firstly, compared with intact cells, the nucleus has the advantage of being easily separated from complex tissues and organs, such as those in the central nervous system. Secondly, snRNA‐seq can be widely used for eukaryotic species, including species from different kingdoms. This method can also provide insights into the regulatory mechanisms of nuclear specificity.^18^ However, it should be noted that snRNA‐seq only captures transcripts in the nucleus, which might fail to capture important biological processes related to, that is, mRNA processing, RNA stability and metabolism. Despite all these potential technical limitations, both technologies have been demonstrated by a large number of scientific publications in, for example, better understanding the cellular and biological processes in organogenesis, gaining novel biomedical and cellular insights into disease pathogenesis.

After the process of converting RNA into the first‐strand cDNA, the resulting cDNA are amplified by either polymerase chain reaction (PCR) or in vitro transcription (IVT).^25^ PCR as a non‐linear amplification process is applied in Smart‐seq,^26^ Smart‐seq2,^27^ Fluidigm C1,^28^ Drop‐seq,^29^ 10x Genomics,^30^ MATQ‐seq,^31^ Seq‐Well ^32^ and DNBelab C4. Currently, there exist two PCR amplification strategies. One uses SMART technology, which takes advantage of transferase and strand‐switch activity of Moloney Murine Leukemia Virus reverse transcriptase to incorporate template‐switching oligos as adaptors for downstream PCR amplification.^33^ This method was the mostly used cDNA amplification method. The other strategy connects the 5′ end of cDNA with either poly(A) or poly(C) to build common adaptors in PCR reaction.^34^ IVT is another amplifying approach and a linear amplification process, which is used in CEL‐seq,^35^ MARS‐Seq,^36^ and inDrop‐seq^9^ protocols. It requires an additional round of reverse transcription of the amplified RNA, which results in additional 3′ coverage biases.^25^ Both approaches can lead to amplification biases. To overcome amplification‐associated biases, unique molecular identifiers (UMIs) were introduced to barcode each individual mRNA molecule within a cell in the reverse transcription step, thus improving the quantitative nature of scRNA‐seq^37^ and enhancing the reading accuracy by effectively eliminating PCR amplification bias. UMIs are adapted by the CEL‐seq,^35^ MARS‐seq,^36^ Drop‐seq,^29^ inDrop‐seq,^9^ 10x Genomics,^30^ MATQ‐seq^31^ Seq‐Well^32^ and DNBelab C4 protocols.^38^

Once the single cell‐barcoded cDNAs are generated from single cells or single nucleus, the cDNA can be sequenced using a number of deep sequencing platforms. In terms of high throughput sequencing based on the DNA nanoballs (DNBseq), the selected DNA fragments was repaired to get a blunt end and modified at the three ends to obtain a dATP overhang, then each end of the DNA fragment was ligated by the dTTP tailed adapter sequence. The ligation product was then amplified for a few cycles, and the following single‐strand cycle was carried out. One special strand of the PCR product was reverse‐complemented with a special molecule and was ligated with the single‐strand molecule by DNA ligase, finally obtaining a single‐strand circular DNA library.^39^ Different scRNA‐seq technology and experimental protocols are summarized in Table 1.

**TABLE 1:** Comparison of different single‐cell RNA sequencing (scRNA‐seq) technology and experimental protocols

Platforms	Isolation strategies	Tissue	Cell numbers	Targets	UMI	Amplification methods	Region	Published year	Reference
Smart‐seq	FACS	Dissociated cell	Hundreds of cells	/	×	PCR	Full‐length	2012	^26^
Smart‐seq2	FACS	Dissociated cell	Hundreds of cells	/	×	PCR	Full‐length	2013	^27^
Fluidigm C1	Micro‐fluidic	Dissociated cell	Hundreds of cells	No poly(A) minus RNA detection	×	PCR	Full‐length	2013	^28^
Drop‐seq	Microdroplets	Dissociated cell	Large number of cell	No poly(A) minus RNA detection	√	PCR	3′ end	2015	^29^
10x Genomics	Microdroplets	Dissociated cell	Large number of cells	No poly(A) minus RNA detection	√	PCR	3′ end	2016	^30^
MATQ‐seq	FACS	Dissociated cell	Hundreds of cells	No poly(A) minus RNA detection	√	PCR	Full‐length	2017	^31^
Seq‐Well	Micro‐fluidic	Dissociated cell	Large number of cells	No poly(A) minus RNA detection	√	PCR	3′ end	2017	^32^
CEL‐seq	FACS	Dissociated cell	Hundreds of cells	No poly(A) minus RNA detection	√	IVT	3′ end	2012	^35^
MARS‐seq	FACS	Dissociated cell	Hundreds of cells	No poly(A) minus RNA detection	√	IVT	3′ end	2014	^36^
inDrop‐seq	Microdroplets	Dissociated cell	Large number of cell	No poly(A) minus RNA detection	√	IVT	3′ end	2015	^9^
DNBelab C4	Microdroplets	Dissociated cell	Large number of cells	No poly(A) minus RNA detection	√	PCR	3′ end	2019	^38^

Abbreviations: FACS, fluorescence‐activated cell sorting; IVT, in vitro transcription; PCR, polymerase chain reaction; UMI, unique molecular identifier; MATQ‐seq, multiple annealing and dC‐tailing‐based quantitative single‐cell RNA‐seq; MARS‐seq, massively parallel single‐cell RNA‐sequencing; DNB‐seq, DNA Nanoball Sequencing .

In conclusion, we would like to highlight questions that should be emphasized in the scRNA‐seq library preparation: (1) how to capture the interesting RNA types from total RNA, also called RNA enrichment; (2) how to reverse transcribe RNA into cDNA fragment with appropriate size; (3) how to connect the adaptor to the end of cDNA. In addition, there are still some remaining challenges to overcome in preparation of scRNA‐seq library. For example, high variability between cells often occurs in scRNA‐seq data, which is caused by technical variations in RNA capture and stochastic transcription in cells.^34^ Furthermore, due to high sequencing cost, previous scRNA‐seq methods only concentrated on the 5′ or 3′ ends of the transcriptome. In fact, the region sample for single cell RNA‐seq should depend on the experimental purpose. For example, even though 3′ end sequencing is cheaper than full‐length sequencing and could provide the best coding region data of 3′ with the addition of a non‐templated poly(A) tail, it cannot sequence the entire tail and cannot specifically report the mRNA isoform to which tails are attached.^40^ In addition, the quality of scRNA‐seq library preparation is affected by other factors, such as technical noise and biological noise. The technical factors include the RNA capture efficiency and quality, random dropouts during library preparation, single‐cell amplification technology and experimental batch effects.^41^ As for biological noise, the nature and different genetic backgrounds of biological specimens (like cell sizes, gene expression) and the dynamic and random environment changes (various cell states, cell cycle states) are difficult to be controlled by experiment operation. Therefore, a still crucial challenge in the scRNA‐seq library preparation is to minimize RNA loss and maximize information Precision.

## 3 STREAMLINE scRNA‐SEQ DATA ANALYSIS

**FIGURE 3:**
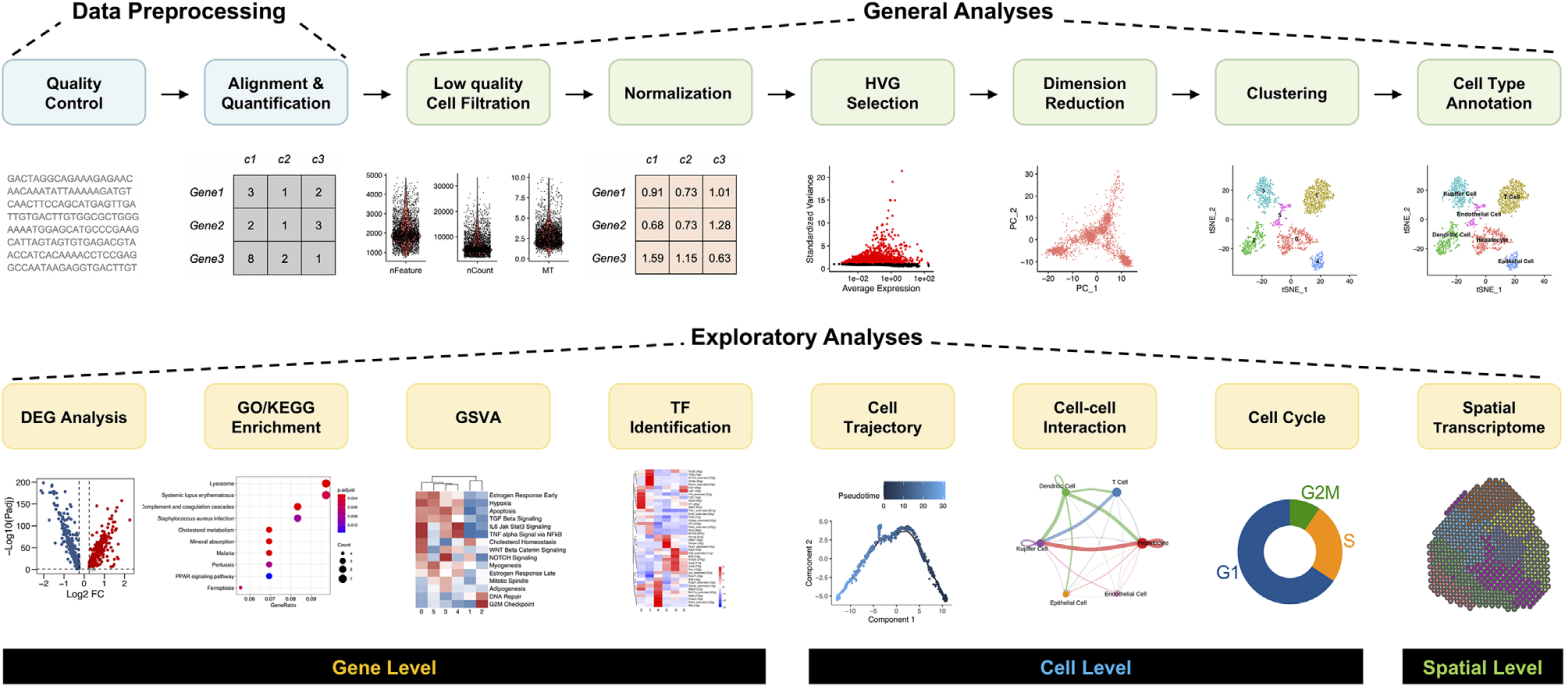
Roadmap for typical single‐cell RNA sequencing data analysis. The classic roadmap for single‐cell RNA sequencing (scRNA‐seq) data analysis mainly consists of data preprocessing (blue panel), general analyses (green panel) and exploratory analyses (yellow panel). Data preprocessing includes quality control, alignment and quantification; general analyses include low‐quality cell filtering, normalization, HVG selection, dimension reduction, clustering and annotation of cell types; exploratory analyses include DEG analysis, function enrichment, GSVA, TF prediction, cell trajectory, cell‐cell interaction, cell cycle and spatial transcriptome analysis. The plot below each box gives a schematic of the visualized results in each analysis step. HVG, highly variable gene; DEG, differentially expressed gene; GSVA, gene set variation analysis; TF, transcription factor. Demo figures were generated with data set GSM4041174

**FIGURE 4:**
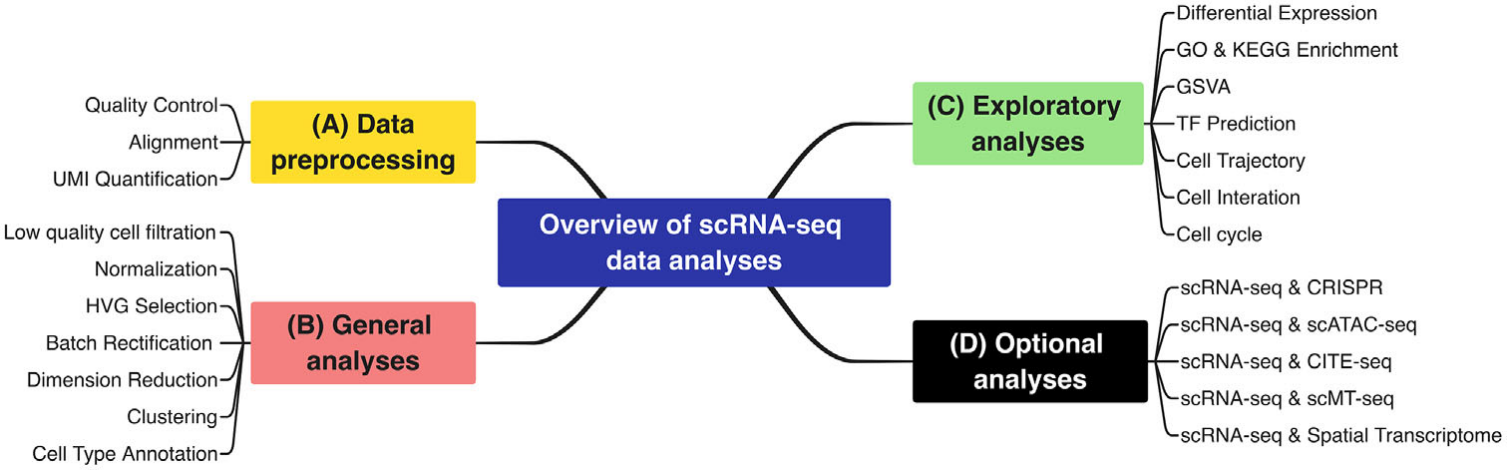
Overview of the analysis modules for single‐cell RNA sequencing data analysis. The diagram shows a summary of analysis modules in the actual analysis of single‐cell RNA sequencing (scRNA‐seq) data, which can be divided into four analysis modules; they are (A) data preprocessing module, (B) general analysis module, (C) exploratory analysis module, and (D) optional analysis module, respectively. More details about each module can be found in the “Streamline scRNA‐seq Data Analysis” section

Analysis of scRNA‐seq data is another key factor, and now the major need, to broaden the application of this technology in life and clinical sciences. To ensure the availability of single‐cell transcriptome analysis tools, numerous developers have made considerable efforts. Nearly 1000 different bioinformatic tools have been developed and made available by May 28th, 2021 (see Table S1).^42^ The increasing number of tools for single‐cell transcriptome analysis has illustrated the importance of analytical methods in the field, but this also means more perplexity in choosing tools for single‐cell data analysis. In this section, we review basic single‐cell transcriptome analysis processes according to key steps (Figure 3), and analysis module (Figure 4), which also covers exploratory analyses of the gene level and cellular level. For advance single‐cell transcriptome data analysis, we refer readers to the specific tools summarized in Table S1 and the original articles reporting the tools.

### 3.1 Data preprocessing

Basic formats of raw sequencing data for single‐cell transcriptome include FASTQ and BCL format, which depend on the data source and sequencing platform. Since only FASTQ files can be directly implemented for quality control, once the raw data are not in FASTQ format, the first step is to convert it to FASTQ format with the appropriate tools. FASTQ files can be generated from the BCL files using *cellranger mkfastq*, a pipeline that has wrapped *bcl2fastq* software. Importantly, a simple CSV matrix file including at least three columns (lane, sample and index) should be provided in addition to the path of BCL files. Then *FastQC* can be applied to assess the quality of raw single‐cell RNA sequencing data.

High‐quality reads need to be mapped to the specific reference genome using an appropriate aligner (e.g., *STAR* or *Tophat*). The *count* is the most important function of *Cell Ranger*, which has wrapped up the alignment, filtering, UMI counting and other practical steps internally. The *Cell Ranger* uses an aligner called *STAR*, which performs splicing‐aware alignment of reads to the genome and then uses transcriptional annotation general transfer format (GTF) file to categorize these reads into exons, introns and intergenic based on whether the reads are aligned to the genome confidently.

### 3.2 General analyses

During the preparation of single‐cell suspension, a viable cell may experience death, cell membrane damage or multicellular adhesion due to unavoidable natural phenomena, experimental operations and technical barriers. To eliminate the gene expression interference from low‐quality cells, it is necessary to conduct a second round of quality control with suitable tools, such as *Seurat*,^43^ *scran* ^44^ *and scanpy*.^45^ In terms of citations, *Seurat* is the most popular one with built‐in functions to handle low‐quality cell filtration. Basically, the following quality control (QC) indicators should be used to judge whether a cell should be retained: the numbers of genes, the numbers of UMI (transcripts), the percentages of mitochondrial genes and the percentages of ribosomal protein genes in each cell. There is no absolute standard for the setting of filter thresholds, which usually depends on the type of cell and tissue being analysed. Lambrechts et al. filtered out cells with ≤ 100 or ≥ 6000 expressed genes, ≤ 200 UMIs and ≥ 10% mitochondrial genes as described in their study.^46^ Fan et al. retained good quality cells using the following parameters: (1) 200 < total number of expressed genes per cell (nGenes) < 2500; (2) 300 < total number of UMIs per cell (nUMIs) < 15000; and (3) percentage of UMIs mapped to mitochondrial genes (MT%) < 10%.^47^ Adjusting the above QC threshold flexibly according to the specific disease state and the diversity of tissue types is recommended. It should be noted the filtration of cells based on mitochondrial genes should be carefully applied as some cell types, such as cardiomyocytes, are biologically more abundant in expressing these genes.

Similar to the analysis of traditional bulk RNA‐Seq data, each cell is treated as an independent sample when analysing the single‐cell RNA sequencing data. The original expression matrix cannot be directly used for downstream analysis because the expression levels between cells are not comparable due to systemic errors or technical noises, such as differences in sequencing depth and transcriptome capture rate for each cell. Normalization is intended to counteract technical noise or bias and to ensure comparability between each cell. In 2020, Lytal et al. evaluated the effectiveness of seven normalization methods, including *BASiCS, GRM, Linnorm, SAMstrt, SCnorm, scran* and *Simple Norm*.^48^ It is worth noting that the speed advantage of Linnorm and scran comes from being written in C++ and implemented in R, which is suitable for large data sets. In contrast, *BASiCS* and *SCnorm* take a longer time to generate more refined results. Overall, there exist large differences among these methods, and different tools perform optimally in different situations.

The single‐cell RNA sequencing data set is high‐dimensional, with tens of thousands of cells in a sample and thousands of genes expressed in each cell. Most genes in each cell belong to housekeeping ones, as they are characterized by no significant changes in the expression level between cells, and their presence tends to obscure the real biological signals. The subsets of features that exhibit high cell‐to‐cell variation in the data set are also called highly variable genes (HVGs). HVGs not only highlight biological signals but also greatly accelerate the downstream analysis of single‐cell RNA sequencing data due to the significant reduction in the computation volume. A high‐quality HVGs should include genes that can distinguish different cell types, and the quality of HVGs has a significant effect on the precision of clustering. In 2018, Yip et al.^49^ evaluated seven methods for detecting HVGs, including *BASiCS, Brennecke, scLVM, scran, scVEGs*, and *Seurat*, observed large differences in the clustering results as well as in the run times of the different methods. Compared with other methods, *scran* can detect a stable number of HVGs with excellent running time and independence from the mean. *Brennecke* was proved to have stable and consistent performances with a wide range of data sets. *scran* and *Seurat* were shown to perform optimally with part of data sets. *BASiCS* and *scLVM_LogVar* were shown to be much slower than others.

Different scRNA‐seq data may arise from different times and different sequencing platforms, and there are inevitable technological or non‐biologically significant batch effects between these data.^50^, ^51^ The batch effect in the scRNA‐seq data has plagued downstream analysis because it can disrupt gene expression patterns and then lead to erroneous conclusions. As a consequence, batch‐effect correction is critical for the analysis of scRNA‐seq data. Although a number of batch effect correction algorithms have been proposed for scRNA‐seq data,^50^ such as *Scanorama* ^52^ and *Seurat V4*,^53^ which can only merge two data sets at a time and integrate multiple data sets by iteration. Most of them consume a large amount of computing memory and time, and this demand is likely to increase as the number of scRNA‐seq data increases. Recently, Zou et al. proposed a novel deep learning‐based method, called *deepMNN*, in order to correct batch effect in scRNA‐seq data.^54^ It compared the performance of *deepMNN* with the most advanced batch correction methods, including the widely used *Harmony*,^55^ *Scanorama* ^52^ and *Seurat V4* ^53^ methods, as well as the recently developed *MMD‐ResNet* ^56^ and *scGen* ^57^ methods based on deep learning. The results^54^ show that the accuracy of deepMNN is better than the existing common methods, especially in the case of large‐scale data sets. And the time complexity and spatial complexity of *deepMNN* algorithm are almost excellent. It took 17 min to complete the batch‐effect correction for the large data set, while *Harmony* and *Scanorama* took about 35 and 77 min, respectively. In addition, it has a larger storage space than *Seurat V4* and *scGen*. At the same time, *deepMNN* can integrate multi batch data sets in one step without multiple iterations. These characteristics of *deepMNN* make it possible to be a new choice for large‐scale single‐cell gene expression data analysis.

In addition to feature selection, dimensionality reduction is also one of the main strategies for processing such high‐dimensional data. For single‐cell RNA sequencing data, two rounds of dimension reduction are generally required, with principal component analysis (PCA) dimension reduction first, and then t‐distributed stochastic neighbor embedding (t‐SNE) or Uniform Manifold Approximation and Projection (UMAP) dimension reduction for visualization. PCA is a mathematical linear dimension algorithm, which uses an orthogonal transformation to transform a series of potentially linearly related variables into new ones that are linearly unrelated, thus using the new variables to show the characteristics of the data at a lower dimension. PCA has been widely used in sRNA‐seq studies to overcome the extensive technical noise in any single feature. Wu et al. conducted a systematic comparison of these two non‐linear dimension reduction methods in 2019. They pointed out the use of UMAP in high‐dimensional cytology and single‐cell RNA sequencing, with particular emphasis on the faster runtimes and consistency of UMAP compared to t‐SNE and the more meaningful organization of cell clusters and preservation of the continuum.^58^ In addition, UMAP has a clear advantage over t‐SNE in the continuity of the cell subsets because it preserves more of the global structure, although t‐SNE is still applied in many single‐cell studies, seemingly due to better visual preferences.

The complexity of single‐cell RNA sequencing data promotes the development of a wide range of clustering methods. Based on the ability to recover known subpopulations, the stability and the run time and scalability, a recent paper^59^ evaluated 14 clustering methods on a total of 12 different data sets. Notably, *SC3* and *Seurat* performed better among these methods in a comprehensive view, with Seurat being several orders of magnitude faster. When the number of clusters was the same*, Seurat* typically achieved the best consistency with the real partition, while FlowSOM achieved better consistency with the real partition if the number of clusters is higher than the real number.

After clustering, assigning a biological annotation to each cluster is the basis of the subsequent analysis. Generally, the workflow for annotating cells in scRNA‐seq data includes three main steps^60^: automatic annotation, manual annotation and validation with wet experiments. Firstly, major automated annotation tools utilize a pre‐defined set of marker genes that are specifically expressed in a known cell type to label clusters by matching their gene expression patterns to known cell types. The advantage of the automated cell annotation method is that it is fast and reproducible, and the results tend to be more reliable in annotating common cell types. However, it is unable to define rare and new cell types due to the limitations of the reference marker gene set. In 2020, Huang et al.^61^ compared and assessed 10 cell‐type annotation methods systematically, including *Seurat, scmap, SingleR, CHETAH, SingleCellNet, scID, Garnett, SCINA, CP* and *RPC*. They found that *Seurat* being the best method for annotating the major cell types among the top five methods: *Seurat, SingleR, CP, RPC* and *SingleCellNet*. However, *Seurat* performed relatively worse in predicting rare cell types and distinguishing highly similar cell types. Secondly, manual annotation is the gold standard method for annotating cells, although it is both subjective and labor‐intensive by searching the relevant literature and mining existing scRNA‐seq data. Finally, wet‐lab experiments are typically required to further validate the finding by scRNA‐seq. Traditional validation methods include immunofluorescence and immunohistochemistry, both of which are based on the principle of specific binding of antibodies to antigens (the surface proteins encoded by marker genes) to prove the true existence of the cell types obtained from the data analysis. Besides, emerging spatial transcriptome sequencing technology can also be considered for increasing the reliability of annotation, which can combine cell imaging and scRNA‐seq to measure spatial transcript patterns and cell morphology in one experiment.^62^

### 3.3 Exploratory analyses

To robustly reveal functional bias and biological significance of specific cell populations, it is necessary to perform functional enrichment analyses on a targeted differentially expressed gene set. Universal analysis strategies for function enrichment are also suitable for single‐cell data, such as gene ontology and Kyoto Encyclopedia of Genes and Genomes (KEGG) pathway. A large number of mature tools for functional enrichment analysis have been developed. Huang et al. comprehensively compared 68 enrichment analysis tools in 2009 after weighing respective advantages and disadvantages.^63^ In addition, *GSVA* is also widely used in functional enrichment analysis and other standard analyses in a pathway‐centered way. *GSVA* can calculate enrichment scores for different signaling pathways in each sample to assess the causes of phenotypic differences, which can be used as a supplement to the KEGG pathway to make the results more biologically explanatory.^64^

To identify the transcription factors enriched in each cell cluster from scRNA‐seq data, Aibar et al.^65^ developed *SCENIC* in 2017, which enabled inferring transcription factors because it firstly achieves the enrichment of TF motifs by searching the putative regulatory regions of target genes. Then TF motif enrichment can realize the connection of candidate TF regulatory factors with candidate target genes. Although *SCENIC* can be implemented in both R and Python, *pySCENIC* is highly recommended for running big data sets due to its faster implementation of the *SCENIC* pipeline. Note that the latest version of *SCENIC* supports *Homo sapiens*, *Mus musculus* and *Drosophila melanogaster*, with the possibility of manually creating a custom database for other species.^65^, ^66^ Although *SCENIC* was broadly used because of its outstanding scalability and robustness for a wide variety of databases, it ignored the dynamic changes in gene regulation mechanisms in different cell types. In 2020, Ma et al. developed IRIS3,^67^ an integrated cell‐type‐specific regulon inference server from single‐cell RNA‐Seq. In practical applications, IRIS3 was more suitable for the researchers without substantial programming skills with its user‐friendly web server. However, continuous improvement is required by IRIS3 in accuracy and efficiency.

Pseudo‐time analysis can be used to infer the trajectory of cells at the single‐cell level, which is expected to discover rare cell types and cryptic states. Different types of analysis tools have been developed in the service of pseudo‐time analysis. In 2019, Saelens et al. conducted a comprehensive comparison of 45 pseudo‐time analysis tools and found great complementarity of existing tools.^68^ *Monocle* is one of the most broadly used tool for pseudo‐time analysis,^69^ which learns an explicit principal graph to describe the data and rebuilds single‐cell trajectories by embedding reversed graph to improve the robustness and accuracy of predicted trajectories. Emphatically, the entire process of establishing single‐cell gene expression kinetics is largely data‐driven.

Organisms will self‐regulate to maintain homeostasis when stimulated, which must require the co‐participation and coordination of multiple cell types. With the rapid development of cell‐cell communication research, the tools available to analyze cell‐cell communication are no longer limited, including *CellChat, CellPhoneDB, NicheNet, SingleCellSignalR* and *iTalk*,^70^, ^71^, ^72^, ^73^, ^74^ etc. Although each of these tools works on the strength of the interaction of ligands and receptors on the cell surface, each has its advantages and weakness. Specifically, if the structural composition of the ligand and receptor is expected to be considered, *CellPhoneDB* should be preferred. If the regulation of cofactors (such as promoters and antagonists) is expected to be taken into account, *CellChat* can be selected to improve performance. It is also recommended to combine multiple cell‐cell communication analysis tools flexibly to avoid methodical bias.

Each cell in the single‐cell suspension is at a specific stage in the cell cycle: DNA synthesis prophase (G1 phase), DNA synthesis phase (S phase), DNA synthesis anaphase (G2 phase) or mitotic phase (M phase). There is a mixture of cells resining different cell cycles from each population. The CellCycleScoring function in the *Seurat* assigns a quantitative score to each cell according to the expression of G2/M and S phase marker genes embedded in its built‐in package. In recent years, machine learning‐based methods have been developed to predict cell cycle stages from single‐cell RNA sequencing data. In 2015, Scialdone et al. compared five established supervised machine learning methods as well as a custom‐built predictor for assigning cells to their cell cycle stages based on the transcriptome. Specifically, they indicate that only PCA‐based methods and customized predictors perform best, which can robustly capture cell cycle signals.^75^

### 3.4 Optional analyses

Although we explained the major steps of the single‐cell sequencing analysis process, there are still many other significant aspects that deserve more attention and exploration, such as the combined application of scRNA‐seq and CRISPR screening,^76^ and the integrated analysis of scRNAseq and multi‐omics, including scATAC‐sEquation (single‐cell chromatin accessibility and transcriptome sequencing),^77^ scMT‐sEquation (single‐cell methylome and transcriptome sequencing),^78^, ^79^ CITE‐sEquation (cellular indexing of transcriptomes and epitopes by sequencing)^80^, ^81^ and spatial transcriptome.^82^ The combination of these techniques enables a better and deeper understanding of key biological processes and mechanisms, which is an important direction for the development of single‐cell technology in the future. In the field of single‐cell RNA transcriptome research, there is still much more potential for analytical algorithms and tools to improve the exploration of data and better understanding of cell functions. Therefore, we also encourage readers to read other excellent reviews that focus on various aspects of scRNA‐seq analysis for more inspiration.^83^

## 4 APPLICATIONS OF SINGLE‐CELL RNA SEQUENCING

**FIGURE 5:**
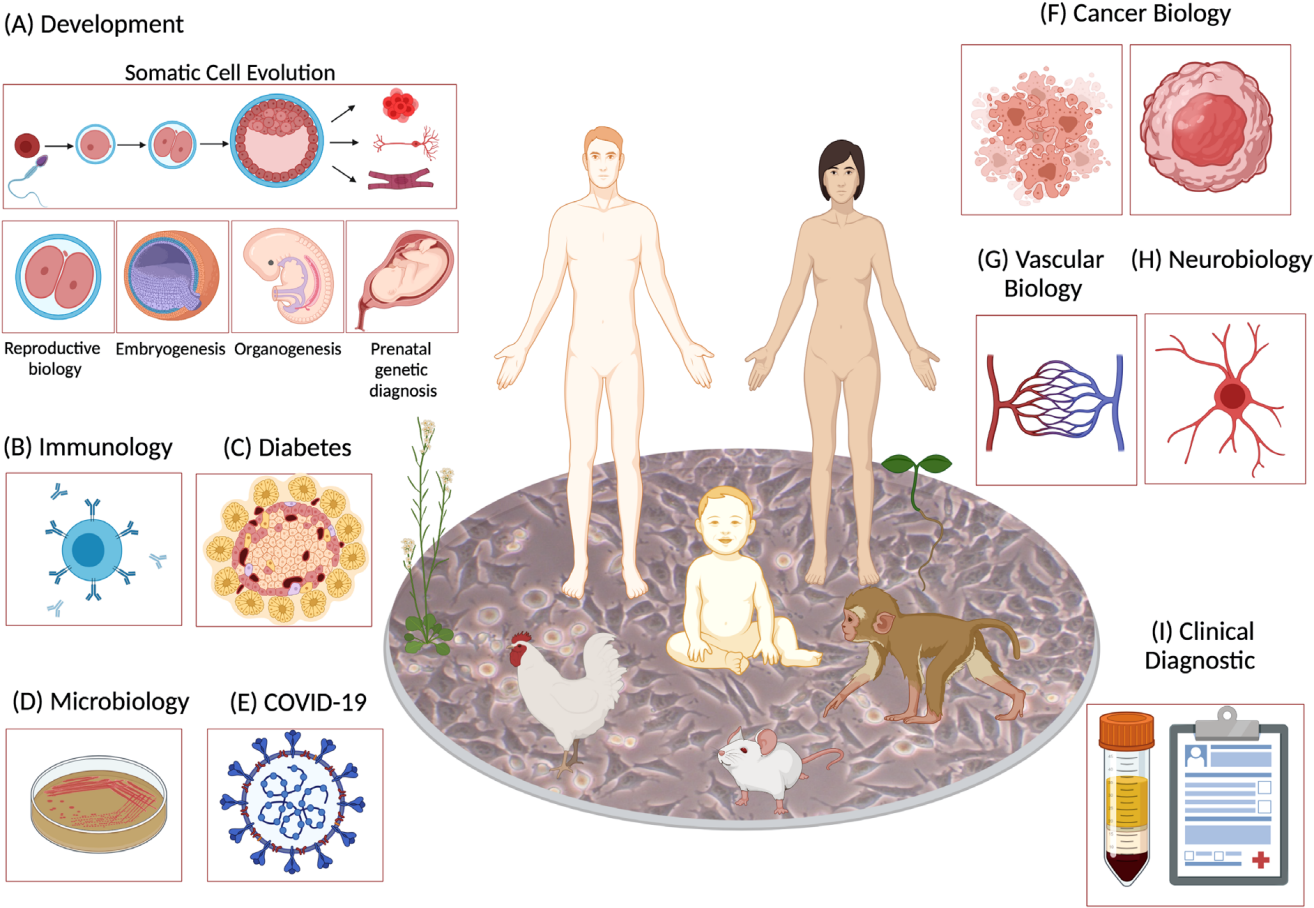
Application of single‐cell RNA sequencing technology. Single‐cell RNA sequencing has been employed in different species (humans, animals, plants) to improve understanding of normal and disease models. A special note is placed on human health, and many single‐cell RNA sequencing (scRNA‐seq) methods are focused on understanding (A) development, (B) immunology, (C) diabetes, (D) microbiology, (E) SARS‐CoV‐2, (F) cancer biology, (G) vascular biology (H) neurobiology and (I) clinical diagnostics. Figure was created with BioRender with license for publication

To date, single‐cell RNA expression profiling is rapidly becoming an irreplaceable method for various research including humans, animals and plants enabling more accurate, rapid identification of rare and novel cells in tissues like never before (Figure 5). Moreover, with the information about gene expression at mRNA and protein levels, metabolites, cell‐cell communication and spatial landscape, it becomes possible to solve the puzzle of cell composition and functions in health and disease. Although the first findings and use of scRNA‐seq were mostly done on animal and later human cells, the sequencing in plant science is still in its early stage and has many exciting challenges remain to be overcome.^84^ To date, the application of scRNA‐seq remains limited to only few plants, due to technical challenges or very limited information on the cell types and discoveries in developmental biology. Several plant research groups used the most used model plant in molecular genetics, *Arabidopsis thaliana* root for high throughput scRNA‐seq and spatial transcriptomics analysis due to the relatively small number of cells, known gene markers and easy methods to isolate individual cells via enzymatic cell wall degradation.^85^, ^86^, ^87^ After successful proof of concept with *A. thaliana* root, the studies have been increasingly applied to the study of other parts of *Arabidopsis* and other plant species such as rice leaf and root, tomato and maize.^88^, ^89^, ^90^, ^91^ Moreover, following the establishment of human cell atlases, the plant‐based scientific community in 2019 initiated a plant cell atlas consortium, aiming to collect more information about various plant cell types, their nucleic acids, proteins and metabolites.^92^ Various web‐based graphical information about plant scRNA‐seq data is available on (https://www.zmbp‐resources.uni‐tuebingen.de/timmermans/plant‐single‐cell‐browser).^93^ Yet, the rapidly growing field of single‐cell biology of the plants has a lot more to offer, including integration of sequencing scRNA‐seq, snRNA‐seq and spatial transcriptomics, imaging techniques and omics will help to further understand changes in genotypes at single cells level.^43^

The scRNA‐seq becomes a powerful tool to profile, identify, classify and discover new or rare cell types and subtypes from different human organs and tissues, giving more profound information about health and disease in development, immunology, diabetes, microbiology, Covid‐19, cancer biology, vascular biology, neurobiology, clinical diagnosis and many other disciplines (Figure 5A‐I). With these new findings unlocking health and disease, we are witnessing rapid progress and changes despite some remaining experimental and bioinformatics challenges. In this section, we highlight and discuss a few important scRNA‐seq applications in biomedical and clinical investigations.

Every tissue/organ contains much morphologically and functionally diverse population of cells in different states, physiological transitions, differentiation trajectories and spatial position. This complex but well‐synchronized microenvironment keeps homeostasis until extreme conditions occur that might turn over the normal cell architecture into, for example, tumours. To understand the initiation of tumours, evolutional origin of cells, tumour progression, metastasis and therapeutic responses, it is important to advance our understanding of tumour microenvironments with essential immune and stromal infiltrates.^94^ A scRNA‐seq analysis can distinguish functionally healthy cells from cancer cells at various developmental stages of tumours. This allows more precise prognoses and diagnoses through the identification and determination of sensitivity to different drugs and develop the most effective treatment strategies for cancers. Initially, scRNA‐seq technology was focused on analysing individual part of the organ, its heterogeneity and cell types involved resulting in producing comprehensive data.^95^ Although there are many scRNA‐seq reports on the individual parts of the tumours, its heterogeneity and cell types involved, but what are the biological functions of each individual cell type, and how the cells talk and work with each other to accomplish their tasks is still largely challenging. The majority of difficulties derive from the fact that tumour tissues are differently positioned in the body; thus their microenvironment contains variety of tumour and non‐tumour cells in different states and stages. Moreover, the cells sample mixture and proportions even within the same section of a tumour might be very different if the biopsy was taken under different times and conditions. In addition, single‐cell gene expression data often contain a lot of noises, and thus cells of the same type might end up in different clusters, and cells of different types can be in the same cluster due to batch effects.^96^ Therefore, it is necessary to carefully sort out high‐quality cell clusters before calculating cell‐type‐specific reference matrix. Although scRNA‐seq is very useful, RNA expression measurements do not always provide information about protein level or post‐translational modifications. Recently, scRNA‐seq studies are supported with other techniques including mass cytometry (cytometry by time‐of‐flight, CyTOF) where for example both studies confirmed that regulatory T cells (T‐reg) in the tumour express higher levels of tumour necrosis factor receptor superfamily member 9 (TNFRSF9; encoding 4‐1BB), inducible T cell co‐stimulator (ICOS) and cytotoxic T lymphocyte‐associated antigen 4 (CTLA4) than T‐reg cells in blood or adjacent normal tissue, possibly reflective of an activated state.^97^ Furthermore, by adding spatial information to scRNA‐seq data, we are able to understand molecular, cellular and spatial tissue organization and cell‐to‐cell interactions in situ.^98^, ^99^, ^100^

Tumour microenvironments are infiltrated with the immune cell types, that is, T lymphocytes cells, CD8^+^ T cells,^101^, ^102^ tumour‐associated macrophages,^103^ cancer‐associated fibroblasts, epithelial cells and cancer stem cells,^104^, ^105^, ^106^, ^107^, ^108^, ^109^ but the types of immune responses and their effects on tumour growth, metastasis and death vary greatly between different cancers and individual tumours.^110^ Immune cells have both anti‐tumour effect inhibiting and killing tumour cells and pro‐tumour activities that promote tumour growths and immune escape.

So far, there are more advanced approaches reported on alterations of immune cells in tumours such as in lung adenocarcinoma,^111^ breast cancer,^112^, ^113^ head and neck squamous cell carcinoma (HNSCC),^114^ nasopharyngeal carcinoma,^115^ head and neck cancer,^116^ pancreatic cancer.^117^ Non‐tumour cells have been investigated starting from the first study on metastatic melanoma where almost 5000 cells both malignant and non‐malignant were analyzed,^105^ following the study led by Lambrechts and colleagues where they identified 52 subtypes of stromal cells, including new subsets of cells. The study found that fibroblasts expressed different collagen proteins, endothelial cells downregulated immune cell homing and genes co‐regulated with established immune checkpoint transcripts and were associated with T cell activity. This study provided a comprehensive map of stromal cell types, and their phenotypes and cooperative behavior, giving a deeper insight into cancer biology that will help advance lung cancer diagnosis and treatment.^46^ In addition, recently Pelka and colleagues developed a systematic approach to apply on two most common type of human colorectal cancer, mismatch repair deficient (MMRd) and MMR proficient (MMRp) tumours, discovering cell types, their underlying programs and cellular communities. They found ‘hotspots of immune activity’ comprised of chemokine‐expressing malignant and non‐malignant cells adjacent to activated T cells.^118^

In tumours, there are active communications between different cell types, including tumour cells, through various signaling pathways. Identifying the communications between tumour and non‐tumour cells will provide important insights into the development of novel therapeutic strategies. There have been also several computational methods developed to infer cell–cell communication from scRNA‐seq data, such as *SingleCellSignalR*, *iTalk* and *NicheNet* that usually use only one ligand‐one receptor gene pairs^71^, ^72^, ^74^, ^119^, ^120^ and *CellPhoneDB* v2.0, which predicts enriched signaling interactions between two cell populations by considering the minimum average expression of the members of the heteromeric complex.^73^ Another platform *CellChat* predicts major signaling inputs and outputs for cells and signals coordinate for functions using network analysis and pattern recognition approaches (http://www.cellchat.org/).^70^

Another important aspect of heterogeneity in tumours that can be investigated by scRNA‐seq is evolutionary process of tumour formation that has been found to play a significant role in the tumour formation as well as acquisition of traits such as chemotherapy treatments and resistance.^121^, ^122^ The continuous accumulation of heterogeneity may reflect the evolution of cancer, and scRNA‐seq can provide meaningful insights into the minor treatment‐resistant cell populations inside complex tumours, which can be used to select appropriate therapies based on tumour type and more precisely treat the individual patient.^123^ Studies on melanoma,^105^ liver cancer^124^ hepatocellular carcinoma,^125^ glioblastoma,^126^, ^127^, ^128^ breast cancer^112^ and prostate cancer^129^ integrate a variety of information in a single cancer cell, deciphering the secrets of cancer heterogeneity and evolution. Furthermore, another emerging technology like spatial transcriptome sequencing incorporates information on the spatial location of cells, providing information on gene expression heterogeneity; organoids can mimic some tumour heterogeneity and 3D organization that can be used for drug screening. All these technologies are indeed needed to properly understand the tumour eco‐system in 3D volume, the role of the endothelial cells in tumour, and how angiogenesis develops in tumours and how tumours react to different treatments. Therefore, vasculature system that compromises the endothelial cells, which line the interior surface of blood and lymphatic vessels plays important roles not only in cancer but also diabetes and neurodegeneration for instance. Endothelial cells play a vital role in maintaining the homeostasis, metabolism and functions of all tissues and organs in the body, such as the exchange of oxygen, nutrients, hormones, fluid and metabolites between bloodstream and surrounding tissues causing various types of dysfunctions. Although this squamous cell is morphological alike in all vessels, there exists a great heterogeneity of functionally distinct phenotypes of endothelial cells. The degrees of endothelial cells heterogeneity have been well recognized for decades.^130^, ^131^, ^132^ For instance, endothelial cells from different vascular beds (artery, capillary and vein), tissues and diseases exhibit substantial heterogeneity. Recently, the heterogeneity of endothelial cells was further extended at the single‐cell transcriptional level. Using single‐cell RNA sequencing, Kalucka et al. profile the single‐cell transcription of over 30 000 endothelial cells from 11 mouse organs. Seventy‐eight endothelial cell phenotypes with distinct transcriptome profile are identified and provide the first murine endothelial cell catalogue for future research.^133^ The degree of endothelial cell heterogeneity can be further illustrated with the increased of cells numbers analyzed. One such example is renal endothelial cells, which is composed of endothelial cells adapted to different physiological conditions in kidney compartments. By analysing over 40 000 renal endothelial cells with single‐cell RNA sequencing, Dumas et al. identified over 20 different phenotypes of endothelial cells in the kidney, which exhibit a high degree of plasticity when exposed to dehydration and hypertonicity conditions.^134^ In adult tissues, endothelial cells are mostly quiescent but metabolically active. However, under pathological conditions such as tumourigenesis, quiescent endothelial cells are activated and involved in the generation of new blood vessels and disease progression. Targeted inhibition of angiogenesis is thus not surprising one of the most broadly used strategies in cancer therapies. However, almost all anti‐angiogenesis therapies in cancers lead to the development of drug resistance, as new mechanisms evolve to replace the drug‐inhibited angiogenesis pathway. Single‐cell RNA sequencing can reveal novel endothelial cell types and mechanisms. By single‐cell sequencing of over 50 000 endothelial cells from lung cancers,^135^ nearly 30 000 endothelial cells from choroidal neovascularization^136^ and over 20 000 from co‐opted breast cancer endothelial cells,^137^ the heterogeneity and transcriptomic/metabolic plasticity of endothelial cells are consistently revealed in these pathological conditions. Most importantly, novel endothelial cell‐targeting targets with therapeutic potential have been identified by single‐cell RNA sequencing approaches.

Another important application of the scRNA‐seq technology is the better understanding of β cell development and pathology in diabetes. Cure of type 1 diabetes (T1D) lies in the restoration of the β cells. However, to generate functional β cells requires extensive understanding of pancreas development, its molecular events and knowledge about the cellular heterogeneity in health and disease. scRNA‐seq studies of developing pancreas were performed firstly on the mouse models, following recent studies on human pluripotent models mostly on embryonic stem cells (ESCs) and induced pluripotent stem cells (iPSCs) 3D models. The studies performed on mouse models revealed several important aspects of the pancreas development. Several groups started on revealing basics in developmental stage identifying a novel α‐cell specific marker Slc38A on wild type model,^138^ another group with Zeng et al. found diverse β‐cell heterogeneity and transcriptomic dynamics during post‐natal maturation and postnatal β cell proliferation. The study identified several novel hallmark features in postnatal β cell proliferation and mass expansion i.e. increased amino acid metabolism, reactive oxygen species (ROS) levels, and *Srf/Jun/Fos* *(* *SRF* *)* transcription factors.^139^ Another group characterized the mechanisms governing pancreatic β and α cells generation, expansion, and maturation during pancreatic development by scRNA‐seq. The study found that the proliferation rate of β‐ and α ‐cells peaks at different developmental time and distinct cell‐type development regulatory pathways were enriched for maturation. Unlike adult β cells, juvenile β cells are more heterogeneous reflecting distinct maturation states.^140^ Other studies deepens the understanding on lineage‐tracing, molecular heterogeneity of precursor cells and identified rare or putative multipotent cells present at stage E13.25 in ductal termini.^141^

The sequence of developmental events is highly conserved between species, for instance, NEUROG3 is transiently and robustly expressed, in two waves, in mice^142^ whereas human NEUROG3 expression occurs in single wave.^143^ The embryonic islet cells of mice are mostly monohormonal, whereas a large proportion of human islet cells are initially polyhormonal.^144^ The scRNA‐seq study confirmed that mouse and human β‐ and α‐cells have differential expression of multiple genes between these species.^145^ These examples highlight the need to confirm any finding obtained in mice in humans. Therefore, the use of the human pluripotent cells is integrated part of developmental biology that can mimic human pancreas development in vitro that has been confirmed in several studies.^146^, ^147^, ^148^, ^149^, ^150^, ^151^

Moreover, 3D cell culture microenvironment more closely resembles in vivo embryogenesis and organogenesis as compared to monolayer (2D) cell culture. This novel approach also increases the functionality of hiPSC‐derived β cells.^152^, ^153^ Notably, 3D organoids are used as a patient‐specific cell model that offer alternative platform to study transcriptomes of T1D. Clustered Regularly Interspaced Short Palindromic Repeats (CRISPR) Cas9 gene editing technology has increased the accessibility of genetically engineered hiPSCs, allowing the manipulation of known or putative regulators of development for their function assessment in human tissues. Furthermore, scRNA‐Seq can be used in combination with CRISPR or lineage tracing.^154^, ^155^ Despite all these great promises, it is important to note that it is particularly challenging to study individual cells in the pancreas due to the high hydrolytic enzyme content of the exocrine cells. Protocols for overcoming these limitations are evolving, including snap‐freezing of the dissected pancreas followed by single‐nucleus RNA‐Seq.^22^ Moreover, single‐cell omics techniques other than scRNA‐seq have been developed such as Patch‐Seq.^156^

In addition to the application of scRNA‐seq in basic life science investigations, this technology has also been demonstrated as a powerful tool for understanding infectious diseases. The COVID‐19 pandemic, caused by coronavirus SARS‐CoV‐2, has affected more than 248 million people worldwide (by 3 November 2021). Understanding the pathogenesis of COVID‐19 infection is of great importance in preventing transmission, reducing the severity of the infection and developing novel therapeutic strategies quickly and efficiently. To date, a number of studies using single‐cell RNA sequencing technology have been conducted to understand the immune cell landscape and response in COVID‐19 patients^157^, ^158^, ^159^ and resulting in variations of clinical outcomes depending on the age, sex, severity and COVID‐19 disease stages.^157^ They found that COVID‐19 induced a unique immune cell signaling in humans compared to healthy controls, particularly during the early recovery phase. Unique immune cell signaling was found in infected humans compared to healthy controls.^158^ Differences in the composition of key immune cells between moderate, severe, convalescent COVID‐19 patients and the control group by performing scRNA‐seq on peripheral blood from COVID‐19 patients and healthy individuals were identified. Most cell types in COVID‐19 patients showed a robust interferon alpha response and an acute immune response.^159^ In addition to single‐cell sequencing of immune cells from peripheral blood mononuclear cells (PBMC) and bronchoalveolar lavage (BAL), single nuclei RNA sequencing of COVID‐19 tissues/organs have also provided important pathological insights in the disease severity and progression. By single‐cell sequencing of 24 lung, 16 kidney, 16 liver and 19 heart autopsy tissue samples and spatial transcriptomics sequencing of 14 lung samples from COVID‐19 patients, Delorey et al.^160^ reveal the biological effects of severe SARS‐COV‐2 infection and remodeling of lung epithelial, immune and stromal compartments in patients. The pandemic is still far from complete dissolution, and scRNA‐seq would certainly remain an important pipeline to properly puzzle immune responses on different variants across the globe. Taken together, single‐cell RNA sequencing technology gained more scientific insights in the fight against COVID‐19 and can be used in the future for detecting not only current SARS‐CoV‐2 but also the other pathogens in combination with conventional methods.

## 5 CONCLUSION REMARKS AND FUTURE PERSPECTIVES

**FIGURE 6:**
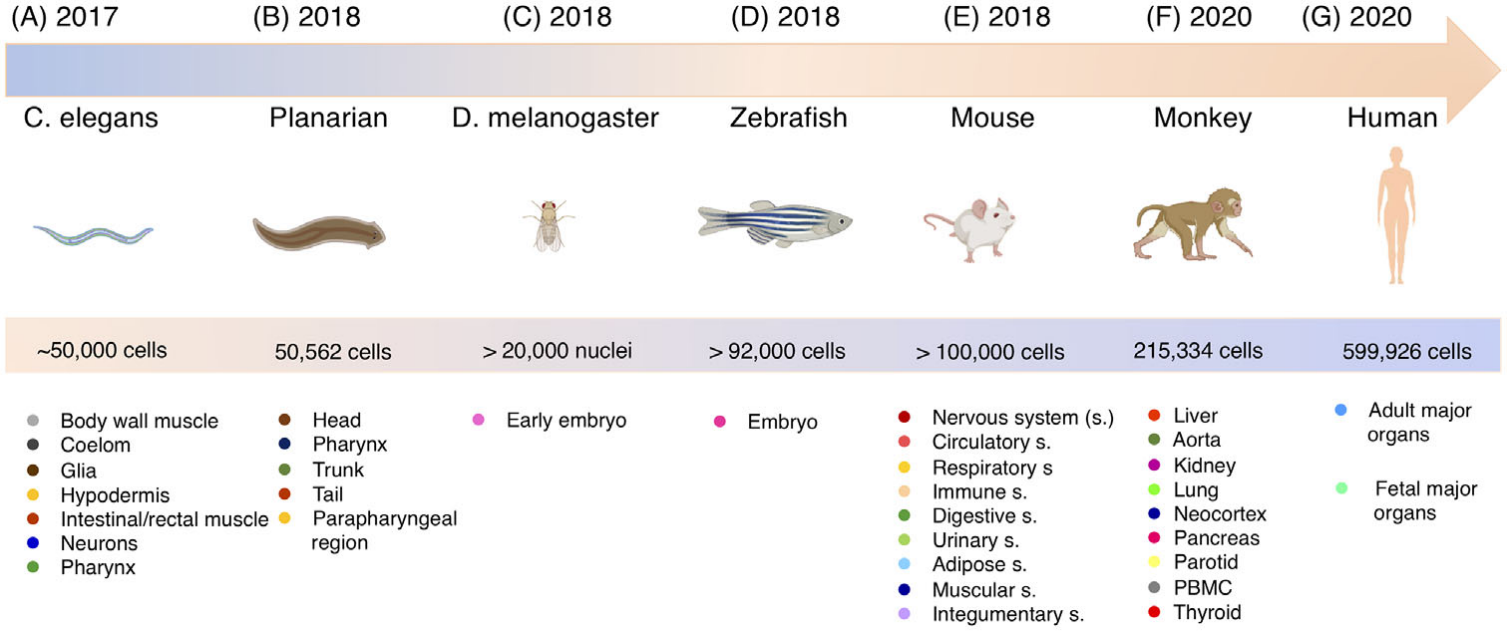
Cell atlases of model organisms. First cell atlases of model organism *Caenorhabditis elegans* (A), planarian (B), *Drosophila melanogaster* (C), zebrafish (D), mouse (E), monkey (F), and human (G). Year of published data, cell number and cell type analyzed by single‐cell RNA sequencing (scRNA‐seq) were indicated. Icons of model organisms are created with BioRender with license for publication

Single‐cell RNA sequencing has proven as one of the transforming technologies in life sciences over the past decade. The development of high throughput single‐cell RNA sequencing technologies and the computational tools make the technology accessible and applicable in almost all applications in life sciences. One important ground knowledge to revisit by using the single‐cell RNA sequencing technology is the construction of single cell atlas in tissues, organs and organism. Towards this, considerable efforts have been made to investigate and establish cell atlases. Just to mention a few, body‐wide single‐cell atlas has been revisited for *Caenorhabditis elegans*, planarian, *D. melanogaster*, zebrafish, mouse, *Macaca fascicularis* and human^7^, ^161^, ^162^, ^163^, ^164^, ^165^, ^166^ (Figure 6). Particularly, Han and coworkers profiled all major human organs, including 60 different human tissue types, and constructed a scheme for the human cell landscape (HCL) for the very first time.^164^ They have uncovered a single‐cell hierarchy for many tissues that have not been well characterized. They profiled more than 599 000 cells using microwell‐seq and established a ‘single‐cell HCL analysis’ pipeline that helps to define human cell identity, genetic networks, progenitor and adult cells. With the development of the technology and applications, more and more single‐cell RNA sequencing data are expected to be generated and integrated in a publicly accessible database to facilitate the understanding gene and cell functions in health and diseases.

Combining scRNA‐seq and other large scale‐genetic screening tools will be further expanding the applications of the technology. One such combinational technology is combing scRNA‐seq and CRISPR‐based genome‐scale genetic screening, such as Perturb‐seq that enables the assessment of transcriptional effects of knocking out several genes with CRISPR,^167^ and LinTIMaT that integrates single‐cell transcriptome data and mutation data for lineage tracing.^168^ In addition to CRISPR‐mediated mutagenesis, it is also possible to combine scRNA‐seq and CRISPR‐mediated gene activation or interference.^169^, ^170^ These combinational applications allow us to investigate the genetic effect on the cellular transcriptome and functions in a large scale. With the continuous development of both single‐cell RNA sequencing and CRISPR gene editing, such as prime editing,^171^ more such combinational technologies and applications are expected to be arrived and contributed to the better understanding of gene and cell functions.

In this review, we focus on the single‐cell RNA sequencing technologies and its applications. However, it should be noted that single‐cell sequencing technology has been developed to measure nearly all OMICS, such as single‐cell whole‐genome sequencing,^172^ single‐cell copy number variation sequencing,^173^ single‐cell epigenetic markers (i.e., DNA methylation, chromatin accessibility) sequencing,^174^ single‐cell proteinomics^175^ and single‐cell metabolomics.^176^ More and more multiomics studies and analyses are expected to be carried out to fully characterize the gene regulatory processes, functions, molecules and interactions for cell types in healthy tissues/organs and in diseased conditions.^177^

Despite all these great promises, one major disadvantage of single cell RNA sequencing is the loss of histological information as both single cell and single nuclei suspensions have to be prepared from tissues. Although trajectory analysis can help with projecting the association and transition between different cell types, other confounding factors associate with tissue digestion, cell isolation and preservation could alter gene expression and cell representation. Spatial dimension of single‐cell transcriptomics represents an essential step and breakthrough in the field to investigate whole organism architecture at the molecular level. Several spatial transcriptomics methods have been developed and demonstrated in proof‐of‐concept studies, such as barcoded array‐based capture of transcripts on microdissected tissues and in situ sequencing. In 2020, spatially resolved transcriptomics technology has been selected as the Method of the Year by Nature Methods. Spatially and temporally revealing the single‐cell transcriptions in a complex tissues and organs will be the rising transforming tools to understand composition, complexity, interaction and functions of cells in tissues/organs/organisms.

Another promising application in the future is integration of the scRNA‐seq technology into routine clinical diagnoses and personalized medicine. However, currently most scRNA‐seq‐based clinical studies are still at their exploratory phases, mainly focusing on revisiting and better understanding the disease processes and identification of diagnosis and therapeutic markers. Although the cost per cell has been reduced significantly, the cost per sample (including the library preparation and sequencing) is still substantially high (Figure 1). This remains one limiting factor to use the scRNA‐seq as a routine diagnostic tool. Other remaining challenges are the scRNA‐seq data processing, analysis, presentation and interpretation. Automatic scRNA‐seq data analysis pipelines with user friendly interphase, and most importantly, which can be used by personnel without any bioinformatic skills and background, are needed to further broaden the scRNA‐seq‐based clinical applications. One such example is the single‐cell omics workbench from the Galaxy Community (https://galaxyproject.org/use/singlecell/), which integrates more than 20 bioinformatics tools. Since a large number of open‐source tools have been developed for this purpose (see Table S1), more streamlined and automatic scRNA‐seq data analysis and visualization platforms are expected to generate and be available in the future. In conclusion, we have presented a brief and concise overview of single‐cell RNA sequencing technology and its applications. The continuous development of the technology will broaden its applications in clinical and personalized medicine.

## CONFLICT OF INTEREST

The authors declare no conflict of interest.

## Supporting information



Supporting Information
